# Reconstruction of the alveolar–capillary barrier in vitro based on a photo‐responsive stretchable Janus membrane

**DOI:** 10.1002/SMMD.20220035

**Published:** 2023-02-21

**Authors:** Changmin Shao, Ting Cao, Xiaochen Wang, Qihui Fan, Fangfu Ye

**Affiliations:** ^1^ Zhejiang Engineering Research Center for Tissue Repair Materials Wenzhou Institute University of Chinese Academy of Sciences Wenzhou Zhejiang China; ^2^ Beijing National Laboratory for Condensed Matter Physics Institute of Physics Chinese Academy of Sciences Beijing China

**Keywords:** alveolar–capillary barrier, in vitro lung models, lung‐on‐a‐chip, photo‐responsive material, stretchable Janus membrane

## Abstract

The lung is the respiratory organ of the human body, and the alveoli are the most basic functional units of the lung. Herein, a photo‐responsive stretchable Janus membrane was proposed for the reconstruction of the alveolar–capillary barrier in vitro. This Janus membrane was fabricated by photocrosslinking methylacrylamide gelatin (Gelma) hydrogel and N‐isoacrylamide (NIPAM) hydrogel mixed with graphene oxide (GO). The Gelma hydrogel containing large amounts of collagen provides a natural extracellular matrix environment for cell growth, while the temperature‐sensitive NIPAM hydrogel combined with GO gives the membrane a light‐controlled stretching property. Based on this Janus membrane, an open polydimethylsiloxane chip was established to coculture alveolar epithelial cells and vascular endothelial cells at the air–liquid interface. It was demonstrated that the alveolar epithelial cells cultured on the upper side of the Janus membrane could express epithelial cell marker protein E‐cadherin and secrete alveolar surfactant. In addition, VE‐cadherin, an endothelium‐specific protein located at the junction between endothelial cells, was also detected in vascular endothelial cells cultured on the underside of Janus membrane. The constructed lung tissue model with the dynamically stretchable Janus membrane is well‐suited for COVID‐19 infection studies and drug testing.

## Graphical abstract

A photo‐responsive stretchable Janus membrane was proposed for constructing a lung‐on‐a‐chip and reconstructing the alveolar–capillary barrier in vitro.

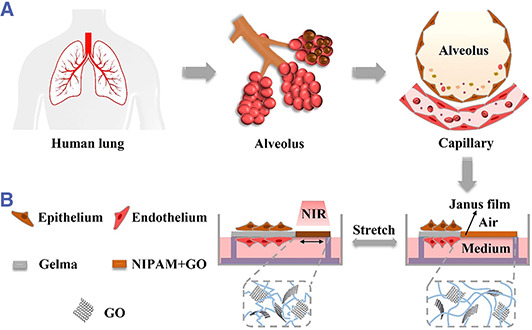

1

**Key points**

- The photo‐responsive stretchable Janus membrane is proposed for the reconstruction of the alveolar‐capillary barrier in vitro.
- An open lung‐on‐a‐chip is prepared based on the Janus membrane to construct the air‐liquid interface of alveoli.
- The constructed lung‐on‐a‐chip with the dynamically stretchable Janus membrane is well‐suited for COVID‐19 infection studies and drug testing.

## 2 INTRODUCTION

The lungs are important organs of the respiratory system responsible for gas exchange between blood and air.^1^, ^2^ In recent years, various chronic respiratory diseases, such as chronic obstructive pulmonary disease (COPD) and asthma, occur frequently, which bring a huge burden to public health.^3^, ^4^, ^5^ However, the lack of accurate in vitro lung models has prevented researchers from studying the progression of human lung disease outside the clinical setting. In vitro cell culture models and mammalian models are still commonly used in studies of the pathogenesis of lung diseases and preclinical drug development.^6^, ^7^, ^8^, ^9^ However, conventional two‐dimensional (2D) cell culture cannot reflect the complex functional characteristics of tissues and organs in vivo nor can it reflect the real response of human tissues and organs to external stimuli.^10^, ^11^, ^12^, ^13^ In addition, although mammalian models can provide certain in vivo information, they also have disadvantages, such as long experimental period, high cost, and large diversity of species.^14^, ^15^, ^16^, ^17^ Consequently, the development of an accurate lung model will provide an effective in vitro platform for disease modeling and drug efficacy evaluation.

In vivo, the alveoli are the most basic structural and functional units of the lung and the main sites of gas exchange in the lung.^18^ Specifically, the alveolar–capillary barrier is primarily constituted by alveolar epithelial cells, vascular endothelial cells, and extracellular matrix (ECM) between them, which shows a significant role in maintaining gas exchange and protecting against external invasion of harmful substances, such as viral infections.^19^, ^20^ To construct the alveolar–capillary barrier in vitro, many attempts have been made to establish the basement membrane for achieving the coculture of alveolar epithelial cells and vascular endothelial cells.^21^, ^22^, ^23^, ^24^ At present, however, most membranes are composed of polyethylene terephthalate, polycarbonate, or teflon, which cannot mimic the properties of ECM.^25^, ^26^ Additionally, to simulate the process of alveolar breathing, sophisticated instruments and complex manipulation are needed to achieve periodic stretching.^27^, ^28^ Therefore, the development of a novel engineered membrane to reproduce the alveolar–capillary barrier and respiratory processes in vitro remains desirable.

**FIGURE 1:**
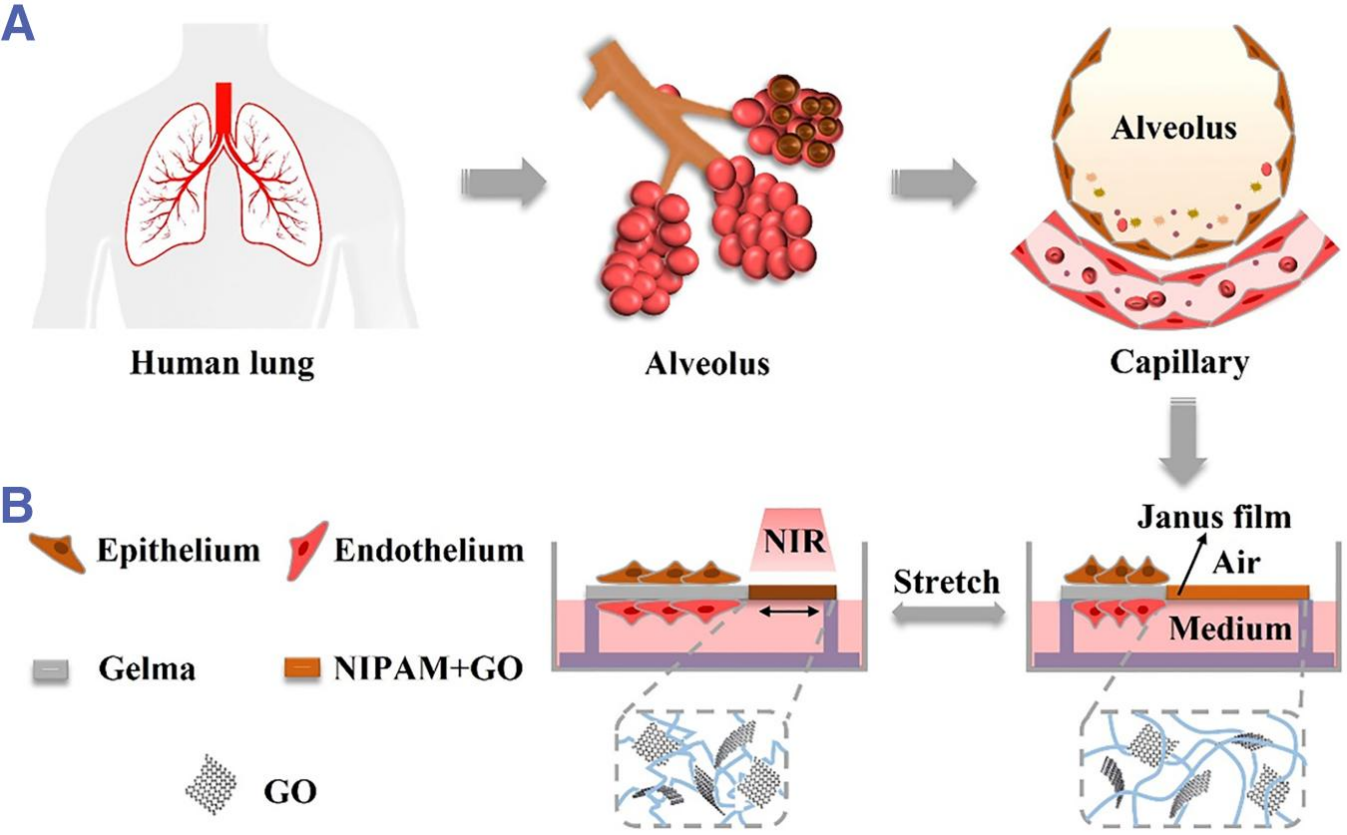
(A) Schematic diagram of the human alveoli. (B) Mechanism of the photo‐responsive stretchable Janus membrane for reconstruction of the alveolar–capillary barrier in vitro.

Herein, we present a novel photo‐responsive stretchable Janus membrane for reconstructing the alveolar–capillary barrier and coculture of alveolar epithelial cells and vascular endothelial cells at the air–liquid interface in vitro, as described in Figure 1. This Janus membrane consisted of two hydrogels, methylacrylamide gelatin (Gelma) hydrogel on one side and N‐isoacrylamide (NIPAM) hydrogel mixed with graphene oxide (GO) on the other. The Gelma hydrogel containing large amounts of collagen provide a natural ECM environment for alveolar epithelial cells and vascular endothelial cells, while the temperature‐sensitive NIPAM hydrogel combined with GO gives the membrane a light‐controlled stretching property. Based on this Janus membrane, a simple open polydimethylsiloxane (PDMS) chip was established to coculture alveolar epithelial cells and vascular endothelial cells at the air–liquid interface. We have demonstrated that the alveolar epithelial cells cultured on the upper side of Janus membrane could express epithelial cell marker protein E‐cadherin and secrete alveolar surfactant. In addition, VE‐cadherin, an endothelium‐specific protein located at the junction between endothelial cells, was also detected in vascular endothelial cells cultured on the underside of Janus membrane. Notably, using this in vitro lung model, we constructed a lung tissue model of COVID‐19 infection and implemented drug testing. These features of our photo‐responsive stretchable Janus membrane make it an ideal material for constructing in vitro lung models.

## 3 RESULTS AND DISCUSSION

**FIGURE 2:**
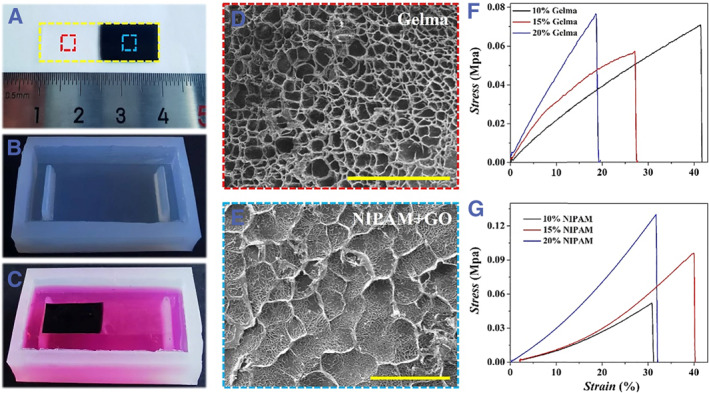
(A) Image of the photo‐responsive stretchable Janus membrane. (B) Image of the PDMS chip. (C) Image of the PDMS chip combined with photo‐responsive stretchable Janus membrane. (D, E) SEM images of Gelma hydrogel (D) and NIPAM + GO hydrogel (E). (F, G) Stress–strain relationship between Gelma hydrogel (F) and NIPAM + GO hydrogel (G) at different concentrations. Scale bars are 50 and 250 μm in D and E. GO, graphene oxide; NIPAM, N‐isoacrylamide; PDMS, polydimethylsiloxane; SEM, scanning electron microscopy.

In this paper, the photo‐responsive stretchable Janus membrane was fabricated using photocrosslinking Gelma hydrogel and NIPAM hydrogel mixed with GO (NIPAM + GO), as shown in Figures 2 and S1. First of all, three glass capillaries with an outer diameter of 350 μm were parallely placed between the two slides. Then Gelma and NIPAM + GO pre‐gel solution were filled at the right and left sides of the center glass capillary, respectively. Finally, the Janus membrane was obtained by removing the center glass capillary and solidifying the two hydrogels by ultraviolet (UV) irradiation (Figure S1A). The generated Janus membrane was observed using optical microscope and scanning electron microscopy (SEM) (Figures 2 and S1). The Janus membrane was 3 cm long, 1 cm wide, and 300 μm thick, and the two hydrogels were evenly distributed on both sides of the membrane (Figures 2A and S1B). The SEM result indicated that Gelma hydrogel exhibited the typical porous structure, and NIPAM + GO hydrogel showed the folded lamellar structure of GO doped in hydrogel (Figure 2D,E).

Gelma is the product of methacrylic anhydride (MA) modification of gelatin and is widely used in tissue engineering due to its excellent biocompatibility and photocrosslinking properties.^29^ In addition, GO is a carbon material with excellent photothermal effect, large specific surface area, and abundant functional groups, which enable it to better combine with polymers to form new composite materials.^30^, ^31^ Moreover, as a temperature‐sensitive hydrogel, the volume of NIPAM will shrink and swell correspondingly with the increase and decrease of temperature.^32^ In conclusion, GO with the photothermal conversion effect can absorb near infrared light (NIR) and rapidly form local high temperature, which triggers the deformation of NIPAM. Thus, the NIPAM + GO hydrogel side of the Janus membrane could quickly respond to the irradiation of NIR light source and deform.

To construct the air–liquid interface of the alveoli, the Janus membrane was integrated into an open PDMS chip (Figures 1, 2 and S1). In simple terms, a simple plastic template with two grooves was generated using 3D printing technology (Figure S1C). Then, the chip with two rectangular convex structures was obtained by copying the template with PDMS (Figure 2B). Finally, the two ends of the Janus membrane were fixed to the rectangular convex with double‐sided tape (Figure 2C).

To optimize the tensile properties of the Janus membrane, the tensile properties of the two hydrogels at different concentrations were tested. The concentrations of the two hydrogels in this experiment were 10%, 15%, and 20% (m/v), respectively. As described in Figure 2F,G, the two hydrogels showed certain elasticity at different concentrations, and the elasticity of NIPAM + GO hydrogel was better than that of Gelma hydrogel. In addition, with the increase in the concentration of the two hydrogels, the elastic modulus exhibited an increasing trend. To achieve periodic stretching, the temperature‐sensitive NIPAM hydrogel and GO with high photothermal conversion efficiency were chosen. Based on our previous findings, the N‐(Hydroxymethyl) acrylamide (NMAM) was mixed into the NIPAM + GO hydrogel to increase the lower critical solution temperature of NIPAM.^32^ Under these conditions, the area shrinkage of NIPAM + GO hydrogel with different concentrations of NIPAM was calculated. As shown in Figure S2, the area shrinkage rate of NIPAM + GO hydrogel decreased with the increase in NIPAM concentration. To sum up, 15% Gelma and 10% NIPAM were selected to fabricate the Janus membrane.

**FIGURE 3:**
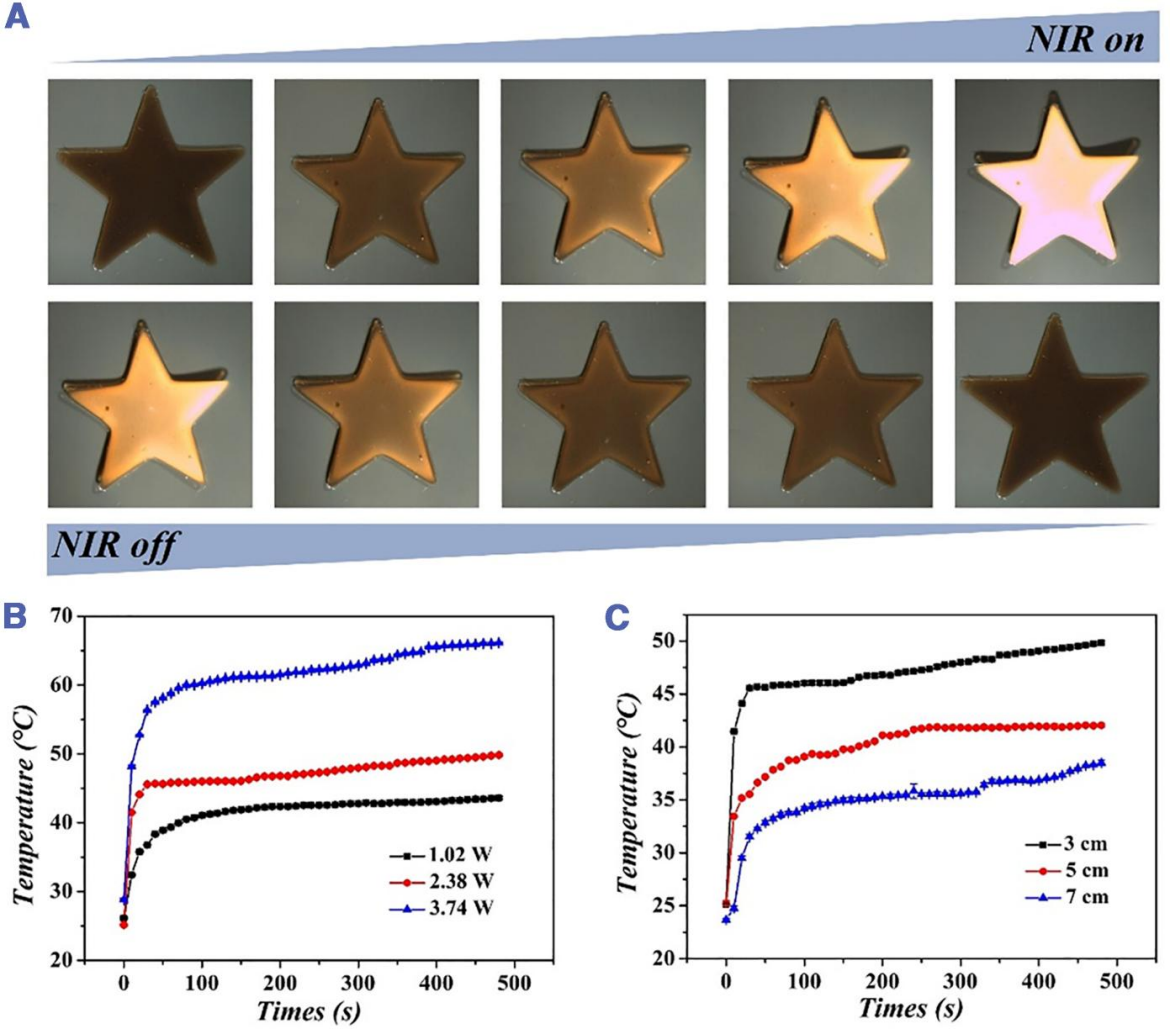
(A) Deformation images of NIPAM + GO hydrogel irradiated by NIR. (B) Relationship between time and temperature of NIPAM + GO hydrogel under different NIR laser intensities. (C) Relationship between time and temperature of NIPAM + GO hydrogel at different NIR radiation heights. GO, graphene oxide; NIPAM, N‐isoacrylamide; NIR, near infrared light.

According to the optimized hydrogel concentration described above, the star NIPAM + GO hydrogel was prepared to visualize the contraction of the hydrogel more visually. As described in Figures 3A and S3, the NIPAM + GO hydrogel shrunk after NIR irradiation and gradually returned to its original state after NIR shutdown. Additionally, the laser intensity and height of NIR radiation were also optimized to observe the temperature changes of NIPAM + GO hydrogel (Figure 3B,C). The results showed that the temperature of NIPAM + GO hydrogel increased as the NIR irradiation power increases or as the height of NIR irradiation light source decreases. Finally, the laser intensity of 2.38 W and the irradiation height of 3 cm were selected for subsequent experiments.

**FIGURE 4:**
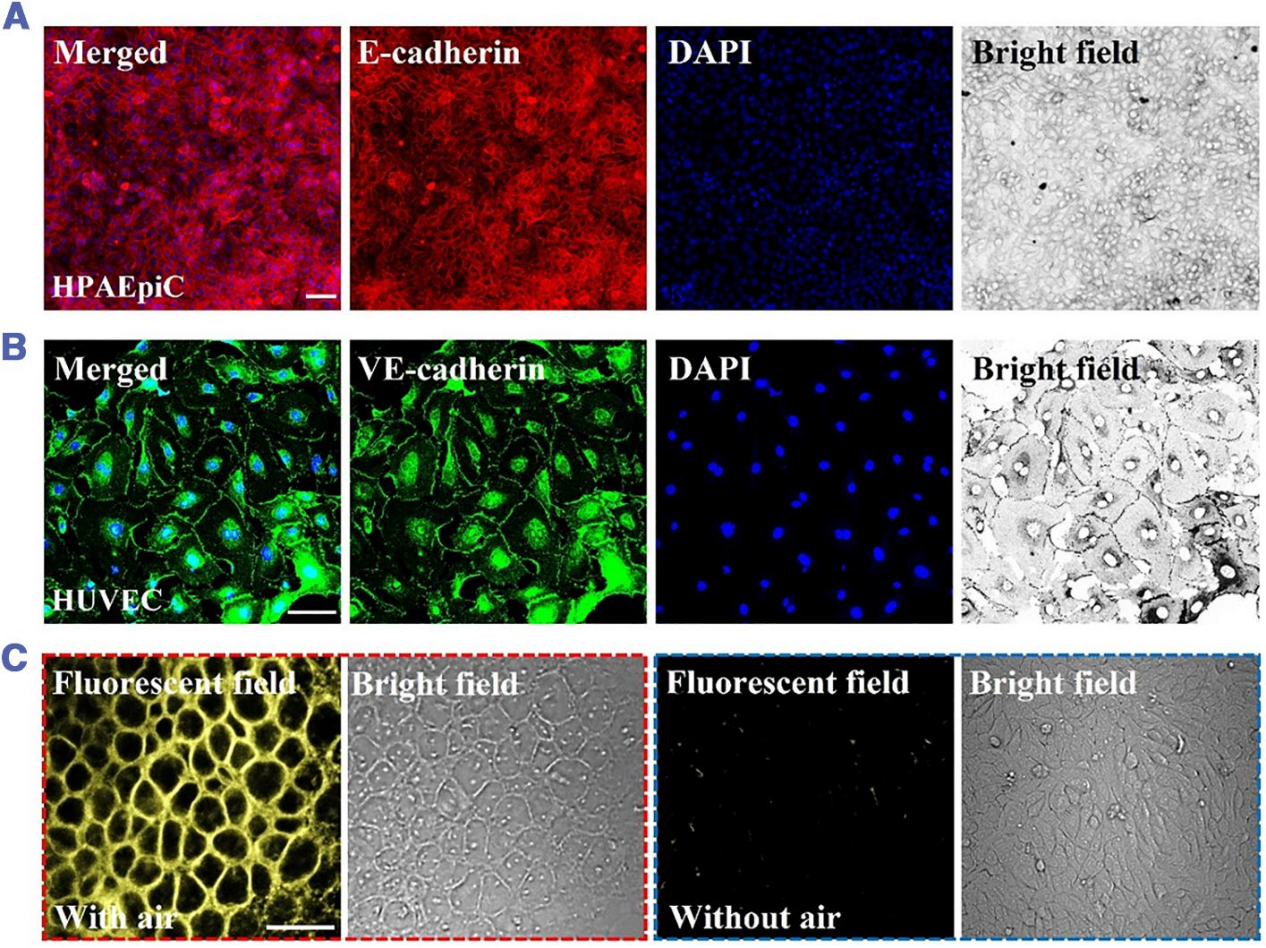
(A, B) Immunofluorescent stain results of HPAEpiC and HUVEC stained with E‐cadherin (A) and VE‐cadherin (B). (C) Results of FM1‐43 staining of HPAEpiC cultured with or without air stimulation. The images were observed by a confocal laser‐scanning microscope (CLSM). Scale bars are 50 μm, 100 μm, and 50 μm in A, B, and C.

To explore the application value of PDMS chip, it was used to construct the in vitro alveolar–capillary barrier. The Gelma hydrogel containing large amounts of collagen provides a natural ECM environment for alveolar epithelial cells (HPAEpiC) and vascular endothelial cells (HUVEC), while the temperature‐sensitive NIPAM hydrogel combined with GO gives the Janus membrane a light‐controlled stretching. Before starting the cell experiments, we first evaluated the biocompatibility of the Gelma hydrogel. The results indicated that HPAEpiC and HUVEC cells showed suitable adhesion and proliferation during coculture with the Gelma hydrogel film (Figure S4). After that, HPAEpiC was seeded onto the upper surface of the Gelma hydrogel film and exposed to air, while HUVEC was cultured on the lower surface of the Gelma hydrogel film and contacted with the culture medium. During cell culture, the cell‐free NIPAM + GO hydrogel area was irradiated with NIR to simulate alveolar stretching. As shown in Figure 4A, HPAEpiC could express the epithelial cell marker protein E‐cadherin, which can regulate intercellular adhesion and maintain the structural and functional integrity of epithelial tissues. Meanwhile, VE‐cadherin, an endothelium‐specific protein located at the junction between endothelial cells, was also detected in HUVEC (Figure 4B).

In vivo, osmophilic lamellar bodies (LB) with diameters of 0.1∼1.0 μm exist in the cytoplasm of alveolar type II cells. LB can secrete alveolar surfactants (dipalmitoyl lecithin) distributed at the air–liquid interface, which reduces alveolar surface tension and stabilizes alveolar size. The alveolar surfactants stained with FM1‐43 dye show bright yellow fluorescence and can be used to monitor the release of surfactants.^33^ As shown in Figure 4C, compared with HPAEpiC without air stimulation, HPAEpiC cultured under air stimulation can produce obvious yellow fluorescence. In addition, the morphology of HPAEpiC was significantly altered in response to air stimulation, producing more intracellular vesicle‐like structures. These results suggest that air stimulation of epithelial cells better mimics the function of the alveolar–capillary barrier in vivo.

To expand the application, the generated chip was used to construct the coronavirus disease 2019 (COVID‐19) model in vitro. COVID‐19 is a new acute respiratory infectious disease caused by SARS‐CoV‐2, which has become a major global public health event. SARS‐CoV‐2 is an enveloped positive‐strand RNA virus that requires fusion with the host cell membrane in order to infect. The spiking (S) protein is the main envelope protein of SARS‐CoV‐2, and its interaction with host cell receptor ACE2 completes the viral infection.^34^ Therefore, the production of antibodies to S protein is essential for host defense against SARS‐CoV‐2 infection. In our experiment, COVID‐19‐pseudovirus was used to infect host cells instead of SARS‐CoV‐2 to build the COVID‐19 model in vitro. The COVID‐19‐pseudovirus containing the S protein of SARS‐CoV‐2 on its surface was developed based on the lentiviral system and contains GFP and luciferase reporter genes. This pseudovirus can effectively mimic the key feature of SARS‐CoV‐2 infection, which is the use of ACE2 as a host cell surface receptor to enter target cells.

**FIGURE 5:**
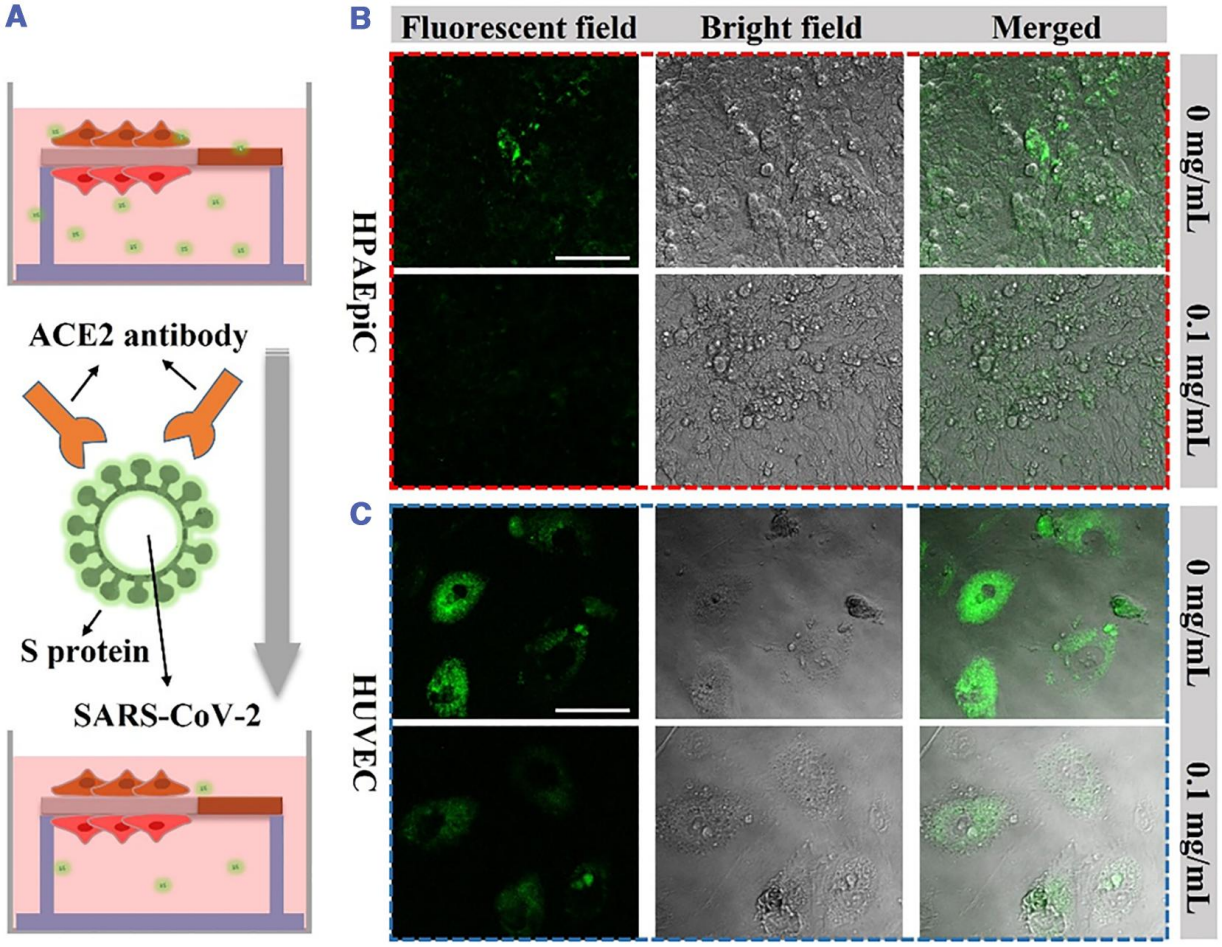
(A) Schematic illustration of the effect of ACE2 antibodies on COVID‐19 infection. (B, C) Effects of ACE2 antibody on HPAEpiC (B) and HUVEC (C) cells infected with COVID‐19 pseudovirus. The images were observed using CLSM. Scale bar is 100 μm.

To sum up, we used the COVID‐19 pseudovirus to infect HPAEpiC and HUVEC to construct the COVID‐19 model and test the effect of ACE2 antibody on COVID‐19 infection (Figure 5A). Our results showed that both HPAEpiC and HUVEC could be infected by the COVID‐19 pseudovirus to produce green fluorescence observed using CLSM. In addition, the intensity of green fluorescence in both cells increased gradually with the increase of pseudovirus concentration (Figures S5 and S6). In subsequent experiments, HPAEpiC and HUVEC cells were infected with pseudovirus at a concentration of 30%. As shown in Figure 5B,C, after the addition of ACE2 antibody, the fluorescence intensity in HPAEpiC and HUVEC cells was significantly reduced. These results indicated that neutralizing antibodies could block the binding of S protein and ACE2, thereby preventing pseudovirus infection of host cells.

## 4 CONCLUSION

In summary, we have proposed a photo‐responsive stretchable Janus membrane for constructing a lung‐on‐a‐chip and reconstructing the alveolar–capillary barrier in vitro. This Janus membrane was fabricated by photocrosslinking Gelma hydrogel and NIPAM hydrogel mixed with GO simultaneously. The Gelma hydrogel containing large amounts of collagen provides a natural ECM environment for cell growth, while the temperature‐sensitive NIPAM hydrogel combined with GO gives the membrane a light‐controlled stretching property. By integrating the Janus membrane into a PDMS chip, lung‐on‐a‐chip was established to coculture alveolar epithelial cells and vascular endothelial cells at the air–liquid interface. It is worth noting that the COVID‐19 model was built using COVID‐19‐pseudovirus to infect alveolar epithelial cells and vascular endothelial cells. We have demonstrated that neutralizing antibodies could block the binding of S protein and ACE2, thereby preventing pseudovirus infection of host cells. Therefore, our lung‐on‐a‐chip based on photo‐responsive stretchable Janus membrane will provide a new platform for constructing lung tissue models and drug testing in vitro.

## 5 MATERIALS AND METHODS

### 5.1 Materials

NIPAM (2210‐25‐5), N, N′‐methylenebisacrylamide (Bis, 110‐26‐9), Gelma (900629), and 2‐hydroxy‐2‐methylpropiophenone (HMPP, 7473‐98‐5) were purchased from Sigma‐Aldrich, USA. NMAM (924‐42‐5) was bought from Aladdin Industrial Corporation. GO (XF0207440‐44‐0) was purchased from XF NANO. HPAEpiC cells were obtained from Mingzhou Biotechnology Co. Ltd.. HUVEC cells and ScienCell ECM 1001 endothelial cell medium were purchased from ScienCell Research Laboratories, Inc.. RPMI 1640 medium (11875119), fetal bovine serum (FBS, 12484028), penicillin–streptomycin double antibiotics (PS, 15070063), 0.25% trypsin‐EDTA (25200072) and L‐glutamine (A2916801) were got from Gibco. PBS (P1020) solution was obtained from Solarbio. CCK‐8 (C0038), live/dead cell double‐staining kit (C2015S) and DAPI (C1005) were obtained from Beyotime Biotechnology. E‐cadherin (mouse mAb, 14472S) and VE‐cadherin antibodies (rabbit mAb, 2500S) were purchased from CST. Goat anti‐mouse Alexa Fluor 647 (ab150115) and goat anti‐rabbit Alexa Fluor 488 (ab150077) antibodies were obtained from Abcam. FM1‐43 (NerveGreen C4, 149838‐22‐2) was bought from US EVERBRIGHT. COVID‐19 pseudovirus (LV‐Spike‐nCoV‐EGFP, 1.67 × 10^7^ TU/mL, LV‐nCov2‐8) was got from PackGene. ACE2 antibody (fc‐hace2) was bought from InvivoGen. Reagents were operated according to the instructions.

### 5.2 Fabrication of the photo‐responsive stretchable Janus membrane

First, three glass capillaries with an outer diameter of 350 μm were placed parallel between the two slides. The 15% (m/v) Gelma pre‐gel solution containing 1% HMPP (v/v) was filled on the right side of the central glass capillary. Then 10% NIPAM (m/v) pre‐gel solution containing Bis (1/29 of the mass of NIPAM), NMAM (1/10 of the mass of NIPAM), GO (2 mg/ml), and 1% HMPP (v/v) was filled into the left side of the center glass capillary. Second, the center glass capillary was pulled out and the two pre‐gels fused together at the junction. Third, the two pre‐gels were solidified by UV irradiation for 40 s. Finally, the Janus membrane sandwiched between two slides was soaked in deionized water and peeled away from the slide.

### 5.3 Establishment of lung‐on‐a‐chip

First, components A and B of the PDMS were mixed in a ratio of 1:1, and the bubbles were removed with a vacuum pump. The PDMS mixture was then poured into the plastic mold with two grooves and cured at 80°C for 3 h. The distance between the two grooves was 3 cm. The PDMS with two rectangular columns was stripped from the mold and treated with plasma for 5 min. Then the two ends of the Janus membrane were fixed to rectangular columns with double‐sided tape. This open PDMS chip integrated with the Janus membrane was disinfected 3 times with 75% alcohol and then rinsed 3 times with sterile PBS before cell culture.

### 5.4 Cell culture

HPAEpiC was cultured in RPMI 1640 medium containing 10% FBS, 1% L‐glutamine, and 1% PS. HUVEC was maintained in ScienCell ECM 1001 endothelial cell medium. The cells were cultured in an incubator containing 5% CO_2_ at 37°C (Memmert, ICO‐150) and digested and passed by 0.25% trypsin‐EDTA when the confluence reached 80%–90%. HPAEpiC with a cell density of 4 × 10^4^ cells/ml was inoculated on the upper side of the Janus membrane. After 4 h of HPAEpiC adhesion, the Janus membrane was turned over and HUVEC with a cell density of 1 × 10^6^ cells/ml was seeded on the lower side of the membrane. Then the Janus membrane inoculated with two kinds of cells was fixed on the PDMS chip with the special double‐sided tape. After that, the cells were cultured in the PDMS chip for 48 h, and the new medium (RPMI 1640:ECM = 1:1) was replaced every 24 h. For the construction of the air–liquid interface, the medium on the upper side of the Janus membrane was removed and HPAEpiC was exposed to air for 12 h.

### 5.5 Cell viability

The cell viability was evaluated using the CCK‐8 assay. HPAEpiC (1 × 10^4^ cells/ml) and HUVEC (5 × 10^5^ cells/ml) cells were cultured on Gelma hydrogel membranes for 1, 3, 5, and 7 days, respectively. The cells in the control group were grown in the 48‐well plates under the same culture conditions. Then 10% CCK‐8 solution was added to each well, and cells were cultured for the following 2 h at 37°C. Finally, OD values were tested at 492 nm with a microplate reader (BioTek EPOCH2NS). Additionally, the cells were stained with Calcein‐AM/PI kit and observed using a fluorescence microscope.

### 5.6 Immunofluorescence staining

First, the cells were fixed at room temperature with 4% paraformaldehyde for 1 h and washed with PBS 3 times for 30 min each time. Then the cells were permeated with 0.5% Triton X‐100 solution for 10 min and washed 3 times with PBS for 30 min each time. After that, 3% bovine serum albumin (BSA) was used to block unspecific proteins for 30 min. Primary E‐cadherin antibody (mouse mAb, 1:50) and primary VE‐cadherin antibody (rabbit mAb, 1:200) were incubated with HPAEpiC and HUVEC cells overnight at 4°C, respectively. Then the cells were washed 3 times with PBS and 30 min for each time. Afterward, the secondary antibodies (goat anti‐mouse 647, 1:150 and goat anti‐rabbit 488, 1:150) were then incubated with the cells at 37°C for 2 h in the dark and washed 2 times with PBS for 20 min each time. Finally, the nuclei were stained for 30 s using 100 nM DAPI and photographed by CLSM.

### 5.7 COVID‐19‐pseudovirus infection

Different concentrations of COVID‐19 pseudovirus prepared with RPMI 1640 were cocultured with cells for 48 h at 37°C. To evaluate the effect of ACE2 antibody on COVID‐19 infection, 30% COVID‐19 pseudovirus and 0.1 mg/ml ACE2 antibody were cocultured with cells. Under the same conditions, the cells without ACE2 antibody served as the control group. The fluorescence expression was observed using CLSM.

### 5.8 Characterizations

The microstructures of the Janus membrane were observed using SEM (Hitachi SU8010). The deformation images of NIPAM + GO hydrogel were recorded using a stereo microscope (Olympus SZX16). The stress–strain curves of Gelma hydrogel and NIPAM + GO hydrogel at different concentrations were characterized by an electronic universal material testing machine (Instron 5944). The fluorescence images of cells were obtained using CLSM (Nikon A1) and inverted fluorescence microscope (ZEISS Axio Vert.A1).

## AUTHOR CONTRIBUTIONS

Fangfu Ye conceived the idea and designed the experiment; Changmin Shao and Ting Cao conducted experiments and data analysis; and Changmin Shao, Ting Cao, Xiaochen Wang, Qihui Fan, and Fangfu Ye wrote the manuscript.

## CONFLICT OF INTEREST STATEMENT

The authors declare no conflict of interest. Fangfu Ye is a member of the *Smart Medicine* editorial board.

## ETHICS STATEMENT

No animal or human experiments were involved in this study.

## Supporting information

Supporting Information S1
