# Supplementary material for: Reconstruction of the alveolar–capillary barrier in vitro based on a photo‐responsive stretchable Janus membrane
Source: Smart Med. 2023 Feb 21;2(1):e20220035. doi: 10.1002/SMMD.20220035 (PMC11235665; doi:10.1002/SMMD.20220035)
Supplement: Supplementary file 1 — Supporting Information S1 [file SMMD-2-e20220035-s001.docx]

**Supporting Information**

**Reconstruction of the alveolar-capillary barrier in vitro based on a photo-responsive stretchable Janus membrane**

Changmin Shao,^1^ Ting Cao,^1, 2^ Xiaochen Wang^1, 2,^ *, Qihui Fan^2,^ *, Fangfu Ye^1, 2,^ *

1. Zhejiang Engineering Research Center for Tissue Repair Materials, Wenzhou Institute, University of Chinese Academy of Sciences, Wenzhou, Zhejiang 325001, China

2. Beijing National Laboratory for Condensed Matter Physics, Institute of Physics, Chinese Academy of Sciences, Beijing 100190, China

***Corresponding author:** [wangxiaochendaisy@ucas.ac.cn](mailto:wangxiaochendaisy@ucas.ac.cn) (X. Wang); [fanqh@iphy.ac.cn](mailto:fanqh@iphy.ac.cn) (Q. Fan); [fye@iphy.ac.cn](mailto:fye@iphy.ac.cn) (F. Ye).


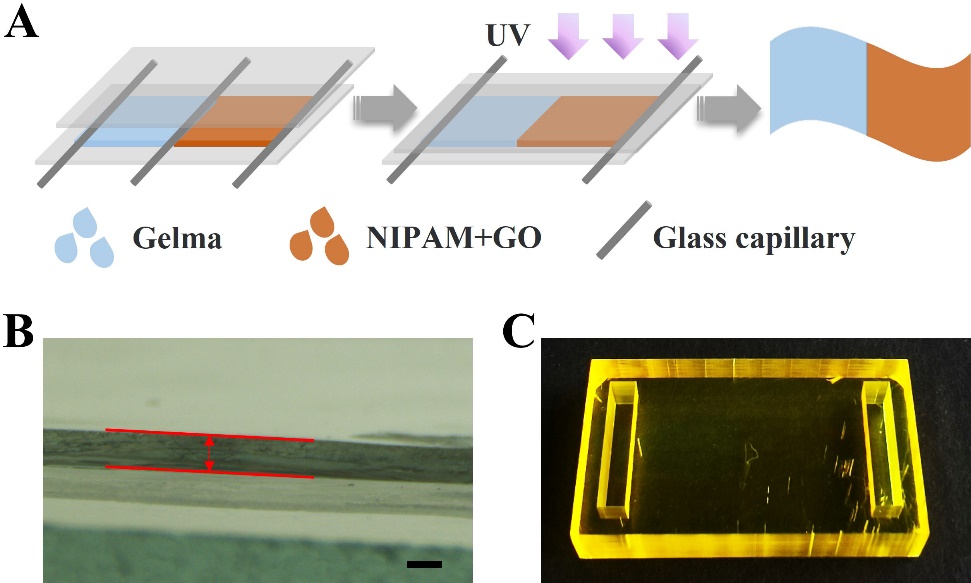


**Figure S1.** (A) Schematic diagram of the preparation of the photo-responsive stretchable Janus membrane. (B) Image of the photo-responsive stretchable Janus membrane. (C) Image of the plastic template with two grooves generated by 3D printing technology. Scale bar is 300 µm in B.


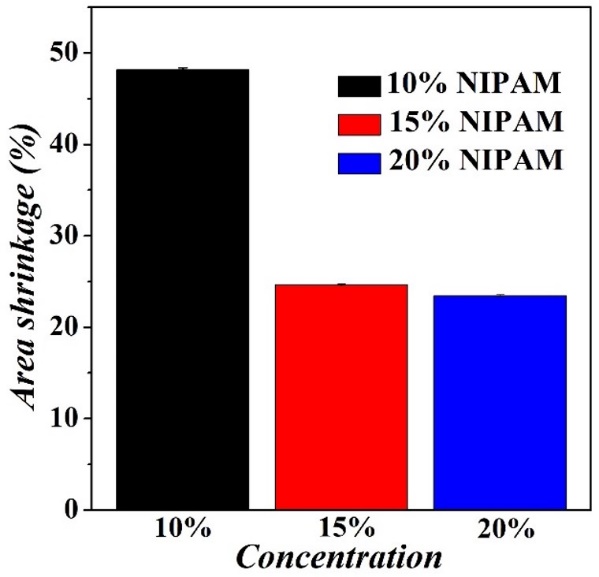


**Figure S2.** Result of the area shrinkage rate of NIPAM+GO hydrogel decreased with the increase of NIPAM concentration.


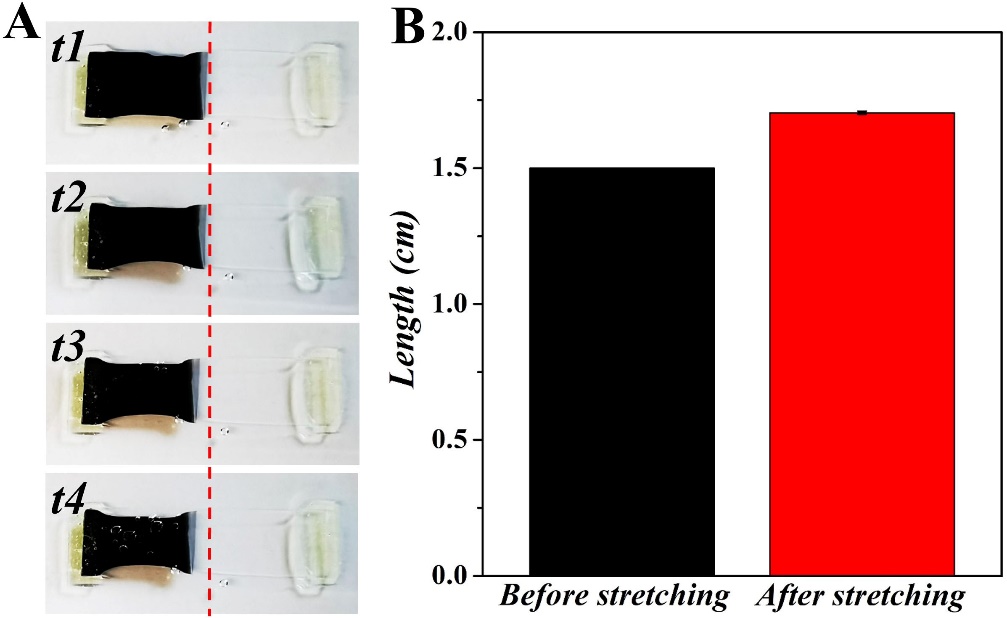


**Figure S3.** (A) Images of the Janus membrane irradiated by NIR. (B) The length of the Gelma hydrogel before and after stretching.


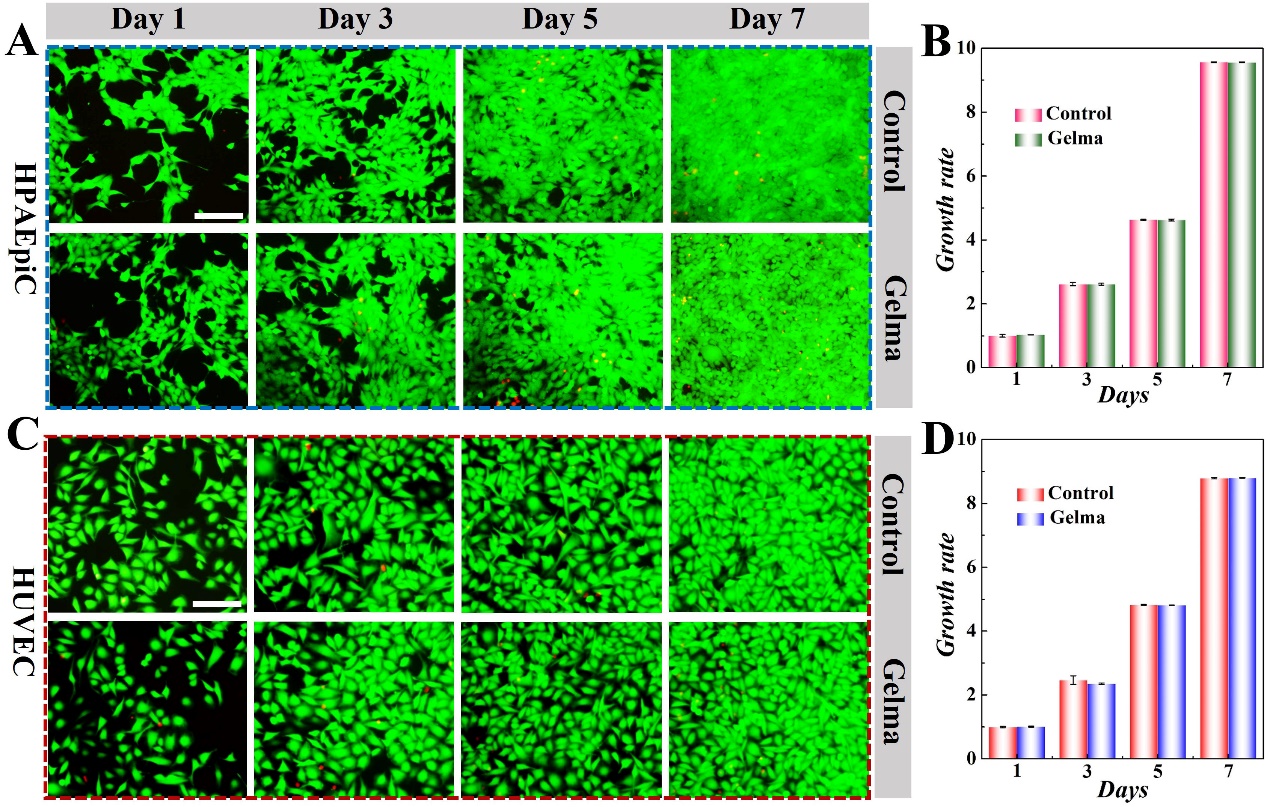


**Figure S4.** (A and C) Images of HPAEpiC (A) and HUVEC (C) cells cocultured with Gelma hydrogel. The cells were stained with Calcein-AM/PI kit and observed by a fluorescence microscope. (B and D) The cell viabilities of HPAEpiC (B) and HUVEC (D) cells cocultured with Gelma hydrogel. Scale bar is 50 µm.


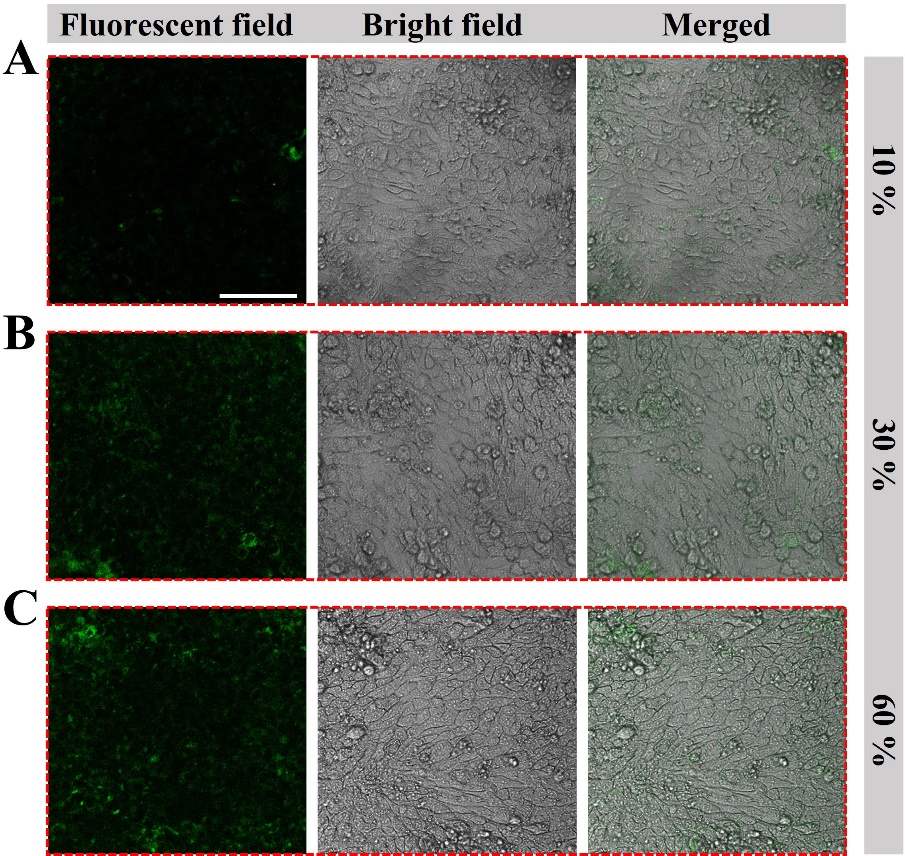


**Figure S5.** Results of infection of HPAEpiC cells with different concentrations of COVID-19-pseudovirus. The images were observed by CLSM. Scale bar is 100 μm.


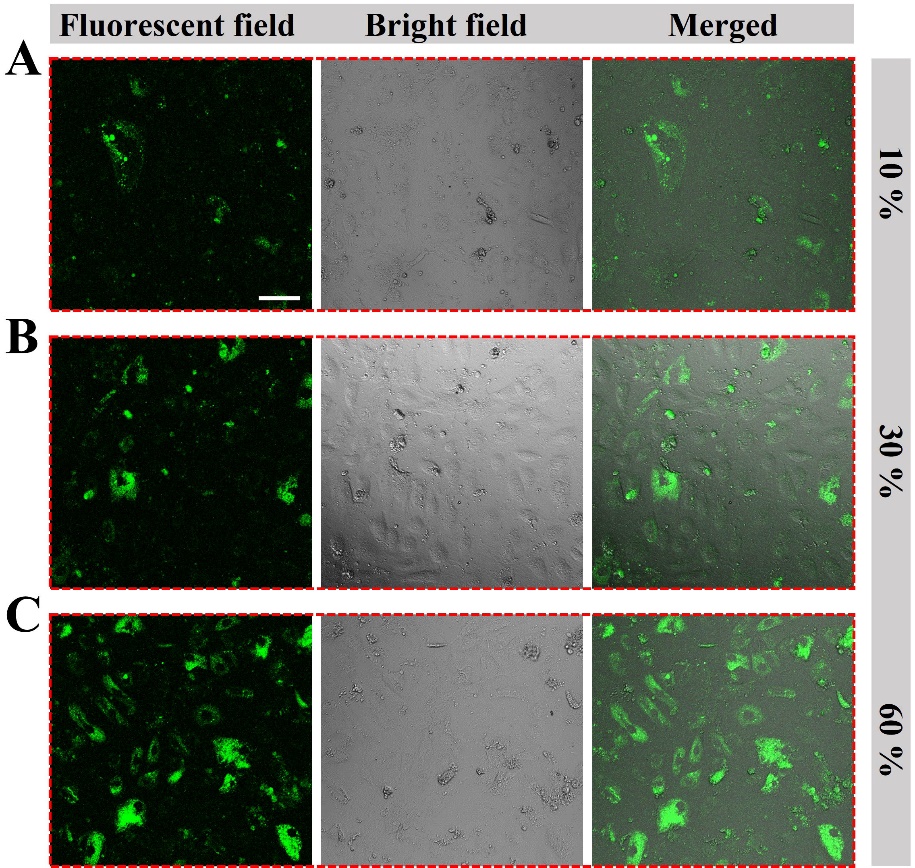


**Figure S6.** Results of infection of HUVEC cells with different concentrations of COVID-19-pseudovirus. The images were observed by CLSM. Scale bar is 100 μm.
